# Cannabinoid Modulation of Excitability and Short-Term Neuronal Dynamics in the Dorsal and Ventral Hippocampus

**DOI:** 10.3390/biology14060642

**Published:** 2025-05-31

**Authors:** Giota Tsotsokou, Ioanna-Maria Sotiropoulou, Klearchos Stampolitis, George D. Oikonomou, Aikaterini-Paraskevi Avdi, Costas Papatheodoropoulos

**Affiliations:** 1 Laboratory of Physiology-Neurophysiology, Department of Medicine, University of Patras, 26504 Patras, Greece; giotatsotsokou@gmail.com (G.T.); str.joanna26@gmail.com (I.-M.S.); klearchosst@gmail.com (K.S.);; 2 Joint Academic Rheumatology Program, First Department of Propaedeutic Internal Medicine, Medical School, National and Kapodistrian University of Athens, Laiko General Hospital, 15772 Athens, Greece; aavdi@med.uoa.gr

**Keywords:** hippocampus, dorsoventral, septotemporal, excitability, inhibition, excitation/inhibition balance, cannabinoid, CB1 receptor, short-term plasticity, rat

## Abstract

Endocannabinoids, acting primarily through CB1 receptors, are critical modulators of neuronal activity, influencing cognitive functions and emotional processing. CB1 receptors are highly expressed in the hippocampus, primarily on GABAergic interneurons, modulating the excitation/inhibition balance. Previous evidence suggests the functional heterogeneity of CB1 receptors along the dorsoventral axis of the hippocampus. However, it is not known whether CB1 receptors differentially modulate basic aspects of the local neuronal network along the hippocampus. This study investigated how CB1 receptor activation modulates excitability, paired-pulse inhibition (PPI), and short-term neuronal dynamics (STND) in the dorsal and ventral CA1 hippocampus under physiologically relevant conditions. Using extracellular recordings from hippocampal slices of male Wistar rats, we compared the effects of two CB1 receptor agonists, ACEA and WIN55,212-2, on network activity in the dorsal and ventral hippocampus. We found that both agonists significantly increased excitability and reduced PPI in the dorsal, but not the ventral, hippocampus. Similarly, CB1 receptor activation modulated STND more prominently in the dorsal hippocampus, reducing facilitation at low frequencies and reversing depression at high frequencies, whereas effects on the ventral region were minimal. These dorsoventral differences in the actions of cannabinoid receptor agonists occurred despite similar CB1 receptor expression levels in both regions, suggesting that functional differences arise from downstream mechanisms rather than receptor density. Pre-application of the GIRK channel blocker Tertiapin-Q occluded the effects of WIN55,212-2 on STND, indicating a significant role of GIRK channel-mediated signaling in CB1 receptor actions. These findings demonstrate that CB1 receptors modulate hippocampal circuitry in a region-specific manner, with the dorsal hippocampus being more sensitive to cannabinoid signaling, likely through differential engagement of intracellular signaling pathways such as GIRK channel activation. These results provide novel insights into how endocannabinoid signaling differentially regulates neuronal dynamics along the dorsoventral axis of the hippocampus. They also have important implications for understanding the role of cannabinoids in hippocampus-dependent behaviors.

## Simple Summary

This study investigates how cannabinoid CB1 receptors affect brain activity in two parts of the hippocampus: the dorsal and ventral hippocampus, which are involved in memory and emotional processing, respectively. Using rat brain slices, we found that activating CB1 receptors by their agonists ACEA and WIN55,212-2 increases excitability, reduces inhibition, and alters the way that principal neurons respond to repeated stimulation in the dorsal hippocampus. In contrast, the CB1 receptor agonists did not significantly alter excitation or inhibition in the ventral hippocampus, and their effects on STND were much less pronounced in the ventral than in the dorsal hippocampus. Interestingly, this dorsoventral difference is not due to the number of CB1 receptors, since both hippocampal segments have similar levels, but rather to downstream signaling mechanisms. Additional experiments suggest that inwardly rectifying potassium (GIRK) channels, which help control neuron activity, contribute to this effect in the dorsal region. These findings show that the endocannabinoid system modulates brain circuits differently along the hippocampus and may help explain how cannabis affects memory and anxiety. Understanding this regional variation could be important for developing targeted cannabinoid-based treatments for neurological and psychiatric conditions.

## 1. Introduction

Endocannabinoids are powerful modulators of brain activity, implicated in a plethora of brain functions, including the processing of cognitive information, learning, memory, reward, and neurogenesis [1]. Furthermore, neuromodulation via endocannabinoids is implicated in pathological conditions such as schizophrenia, depression, anxiety, Alzheimer disease, and multiple sclerosis [1,2,3,4,5]. Endocannabinoids mediate their actions through two types of cannabinoid (CB) receptors, type 1 (CB1 receptors) and type 2 (CB2 receptors), which are G-protein-coupled receptors. CB1 receptors are distributed in the central nervous system and influence neuronal activity by modulating the activity of other neurotransmitter receptors and ion channels [6,7,8]. CB2 receptors are predominately expressed in immune cells [9], but they also mediate significant functions in the brain, acting complementary to CB1 receptors [10]. CB receptors are primarily coupled to inhibitory G proteins (G_i/o_), but they also engage other signaling pathways, including G_q/11_ pathways, and they inhibit adenyl cyclase and inwardly rectifying potassium (GIRK) channels [6,7,8]. Through these actions, CB1 receptors play significant roles in modulating synaptic transmission, neuronal excitability, and synaptic plasticity [8,11].

CB1 receptors are widely expressed in the brain and found in various subcellular compartments, with especially high expression in presynaptic terminals [12,13,14]. The prominent presynaptic expression of CB1 receptors in combination with the postsynaptic synthesis of endocannabinoids has led to the established assumption that endocannabinoids control synaptic transmission through retrograde signaling mechanisms involving CB1 receptors [8,15,16]. However, CB1 receptors are also found postsynaptically, where they influence neuronal excitability, synaptic plasticity, and long-term neuronal adaptation [15,17,18]. CB2 receptors can also influence behavior by modulating neuronal excitability [10,19,20].

The hippocampus contains high levels of CB1 receptors [13], predominately on the axon terminals of GABAergic interneurons and, to a lesser extent, excitatory glutamatergic terminals [21,22], controlling GABA and glutamate release [23,24]. Through this localization, the activation of CB1 receptors in the hippocampus suppresses inhibitory GABAergic neurotransmission more than excitatory glutamatergic transmission [25], thereby modulating the excitation/inhibition (E/I) balance in the network [25,26,27]. However, by affecting the E/I balance, the activation of CB1 receptors can disrupt the organization of neuronal firing and affect network oscillations in the hippocampus, such as the theta rhythm [28]. This disruption may impair hippocampus-dependent functions such as spatial learning and memory [29]. In addition, cannabinoids (CBs) strongly modulate anxiety-like behaviors and emotional processing [30,31]. Notably, spatial learning and memory predominately engage the dorsal hippocampus, while anxiety primarily involves the ventral hippocampus [32,33]. Considering that the E/I balance undergoes distinct constitutive fine-tuning in the two segments of the hippocampus [34], the actions of CBs may occur through differential regulation of the E/I balance in the two segments. For example, recent evidence suggests that CB1 receptors are involved in the strongly heightened network excitability associated with reduced inhibitory and excitatory transmission induced by the co-activation of NMDA and metabotropic glutamate receptors in the dorsal hippocampus [35]. However, it is not yet clear whether CBs influence the E/I balance differently in the dorsal and ventral hippocampus under more physiological contexts.

One of the most well-known actions of CB receptors in modulating neuronal circuit function is the depolarization-induced suppression of inhibition or excitation, a phenomenon associated with a transient reduction in transmitter release mediated by retrograde signaling of endocannabinoids following the depolarization or firing of postsynaptic neurons [36,37,38,39,40]; for a review, see [16]. Endocannabinoids also mediate a short-term depression of inhibitory and excitatory synapses following the activation of postsynaptic group I metabotropic glutamate receptors [41]. Though these phenomena are related to the actions of presynaptic CB1 receptors on synaptic transmission, how endocannabinoids can transiently modulate the output of principal cells under conditions of repeated neuronal activation has not yet been sufficiently clarified. Notably, in addition to their presynaptic effects, CBs can modulate neuronal activity through postsynaptic mechanisms. For instance, evidence shows that CB1 receptors activate GIRK channels [42,43] and hyperpolarization-activated cation channels (I_h_) [44] and can directly modulate extrasynaptic GABA_A_ receptors [45].

The transient changes in neuronal firing following high-frequency activation (i.e., short-term dynamics, STND) is a physiological phenomenon involving transient changes in GABAergic transmission and E/I balance [46,47] and is implicated in neural information processing such as firing pattern detection associated with the temporal coding of stimulus properties [48] and the dynamic regulation of network excitability associated with the prevention of network hyperexcitability [49]. Interestingly, STND displays a striking dissociation between the dorsal and the ventral hippocampus, with the dorsal hippocampus displaying frequency facilitation and the ventral hippocampus displaying mostly frequency depression [47,50,51].

Considering these dorsoventral differences and that CBs can modulate synaptic inhibition and E/I balance, we hypothesized that CB receptors may distinctly shape STND in the dorsal and ventral hippocampus by exerting differential effects on the E/I balance in these two segments. Therefore, we conducted a comparative investigation into the effects of cannabinoid receptors activation on excitability, paired-pulse inhibition (PPI), and short-term neuronal dynamics (STND) at the Schaffer collateral CA1 synapses between the dorsal and ventral hippocampus using extracellular recordings. Our findings indicate that CB1 receptors play a crucial role in regulating excitation, PPI, and STND in that dorsal more than in the ventral hippocampus, despite similar CB1 receptor expression in the two hippocampal segments. Furthermore, our results suggest a partial involvement of GIRK channels in the modulation of STND through cannabinoid signaling, highlighting a potential mechanism underlying region-specific differences in hippocampal processing.

## 2. Materials and Methods

### 2.1. Experimental Animals and Hippocampal Slice Preparation

This study utilized adult male Wistar rats raised under controlled environmental conditions (21 ± 1 °C, 12-h light/dark cycle) at the Animal Facility of the Medical School, University of Patras (license EL-13-BIOexp-04). All experimental procedures adhered to the European Directive 2010/63/EU on animal welfare and were approved by the institutional Research Ethics Committee and the Regional Directorate of Veterinary Services (approval no. 5661/37, 18 January 2021). The required sample size was calculated using G*power software, version 3.1.9.7. To avoid sex-related variability in neuronal responses to CB1 receptor activity, we restricted our experiments to male subjects. This decision was based on evidence suggesting that cannabinoid receptor signaling is modulated by the estrous cycle [52,53], potentially confounding the interpretation of comparative regional effects within the hippocampus. For instance, in the present study, we found relatively modest effects of CB1 receptor agonists in the ventral hippocampus of male animals. A weaker or absent effect on the ventral hippocampus of female animals could have masked this action, leading to the erroneous conclusion that CB1 receptor agonists are ineffective in the ventral hippocampus. Nonetheless, we consider it necessary to perform a future comparative study of cannabinoid actions on short-term plasticity phenomena in the hippocampus between sexes.

After deep anesthesia and decapitation, brains were rapidly extracted and immersed in chilled (~4 °C) artificial cerebrospinal fluid (ACSF) composed of (in mM) 124 NaCl, 4 KCl, 2 CaCl_2_, 2 MgSO_4_, 26 NaHCO_3_, 1.25 NaH_2_PO_4_, and 10 glucose, adjusted to pH 7.4 and continuously bubbled with 95% O_2_ and 5% CO_2_. Hippocampi were isolated, and 500 μm thick transverse slices were obtained from dorsal and ventral regions (0.5 to 3.5 mm from each end) using a McIlwain tissue chopper as previously described [54]. Slices were transferred to an interface recording chamber, continuously perfused with oxygenated ACSF (~1.5 mL/min) at a stable temperature of 30 ± 0.5 °C, and allowed to recover for at least 90 min prior to recording.

### 2.2. Electrophysiology and Data Analysis

Population spike (PS) responses were evoked in the CA1 pyramidal cell layer by stimulating Schaffer collaterals using a bipolar platinum/iridium electrode (25 μm wire diameter, 100 μm spacing; World Precision Instruments, Friedberg, Germany). Recording was performed with a 7 μm thick carbon fiber electrode (Kation Scientific) positioned 200–400 μm from the stimulation site. Electrical pulses were delivered every 30 seconds, and signals were amplified (×500), filtered (0.5 Hz–2 kHz), digitized at 10 kHz, and stored using Signal 5.9 software and a CED 1401-plus interface (Cambridge Electronic Design, Cambridge, UK). Only slices with stable PS amplitudes over a 10 min baseline were used for analysis. Data from slices showing >10% fluctuation or experimental artifacts were excluded. Input–output curves relating stimulus intensity to PS amplitude (measured from the negative peak to the baseline line connecting adjacent positive peaks) were constructed. The amplitude of the PS was measured as the distance from the minimum peak to the line connecting the two maximum peaks of the PS waveform.

To examine STND, we applied ten-pulse stimulus trains at varying frequencies (0.1 to 100 Hz) in a random order, with 2 min intervals between trains. The stimulation intensity was initially set to evoke PS amplitudes between 0.5 and 2.0 mV under drug-free conditions. Frequency-induced changes were quantified by averaging the percentage changes of conditioned responses (PS2–PS10) relative to the initial response (PS1) within each train [47]. When pharmacological agents altered the amplitude of PS1, we repeated the frequency protocol with adjusted stimulus intensity to match pre-drug PS1 levels since previous studies reported that the magnitude of the conditioning PS (PS1) significantly influences the profile of STND [47]. To some extent, this is expected since a higher PS can lead to more intense recurrent activation of the local network of inhibitory interneurons, which are involved in determining the profile of STND [47]. Therefore, experimental conditions that affect PS amplitude may influence STND both directly (e.g., by affecting neuronal excitability) and indirectly by altering the excitatory drive of GABAergic inhibition. Additionally, some experimental conditions may directly modify inhibitory mechanisms. Thus, this methodological step helped distinguish between direct drug effects on STND mechanisms and indirect effects resulting from altered network excitability, including recurrent inhibition. For example, in the dorsal hippocampus, CB1 receptor activation was found to enhance PS amplitude and reduce inhibitory tone. To address both direct and indirect contributions, STND was quantified in both the non-adjusted and adjusted conditions, and their average was used for statistical comparisons.

### 2.3. Immunoblotting

CA1 regions from the dorsal and ventral hippocampus were excised from 500 μm thick slices and homogenized in 200 μL of 1% SDS containing protease inhibitors (Sigma Aldrich, Taufkirchen, Germany) using sonication. Protein concentrations were determined using a NanoDrop™ 2000 spectrophotometer (Thermo Scientific, Waltham, MA, USA). Equal amounts of protein (25 μg per lane) were loaded onto 10% SDS-PAGE gels and subjected to electrophoresis (30 min at 80 V, 1 h at 120 V). Proteins were transferred to PVDF membranes at 400 mA for 90 min. Blocking was performed in 5% non-fat milk in PBST (PBS + 0.01% Tween-20) for 1 h at room temperature. Membranes were incubated overnight at 4 °C with primary antibodies diluted in 3% PBST: rabbit anti-CB1 (1:1000; Abcam, Cambridge, UK; ab259323) and mouse anti-beta-actin (1:10,000; ThermoFisher, MA5-11869). After rinsing, membranes were incubated with HRP-conjugated secondary antibodies (anti-rabbit or anti-mouse IgG) for 1 h at room temperature. Bands were detected using enhanced chemiluminescence (ECL; Millipore, Darmstadt, Germany; WBULS0500) with a ChemiDoc MP system (BioRad, Hercules, CA, USA), and densitometry was performed using ImageLab 6.1 software. CB1 protein expression was normalized to beta-actin (relative optical density, ROD), and data were analyzed statistically.

### 2.4. Drugs

The following drugs were used in this study: the potent and highly selective agonist of CB1 receptors N-(2-Chloroethyl)-5Z,8Z,11Z,14Z-eicosatetraenamide (ACEA), the potent aminoalkylindole cannabinoid receptor agonist [(3R)-2,3-dihydro-5-methyl-3-(4-morpholinylmethyl)pyrrolo[1,2,3-de]-1,4-benzoxazin-6-yl]-1-naphthalenyl-methanone, monomethane sulfonate ((+)-WIN 55,212-2 (mesylate)), the potent and selective CB1 receptors antagonist/inverse agonist 1-(2,4-dichlorophenyl)-5-(4-iodophenyl)-4-methyl-N-4-morpholinyl-1H-pyrazole-3-carboxamide (AM281), and the high-affinity blocker of inward-rectifier K+ channels Tertiapin-Q (50 nM), which blocks GIRK channels (Kir1.1, Kir3.1, and Kir 3.4 inwardly rectifying potassium channels). ACEA and Tertiapin-Q were purchased from Tocris Cookson Ltd., Bristol, UK; WIN55,212-2 and AM281 were purchased from Cayman Chemical Company, Ann Arbor, MI, USA.

### 2.5. Statistics

Throughout the text and figures, values are expressed as mean ± S.E.M. The number of slices and animals is given (slices/animals). IBM SPSS Statistics 27 software package was used for performing independent *t*-tests to compare values between the two poles of the hippocampus and paired *t*-tests to determine drugs’ effects. Population variances were examined for equality using the Levene’s test. The normality of the distribution of the values for the various variables was assessed using the Shapiro–Wilk test. Corresponding t or z values were reported depending on whether the data were normally distributed or not.

## 3. Results

This section may be divided by subheadings. It should provide a concise and precise description of the experimental results, their interpretation, as well as the experimental conclusions that can be drawn.

### 3.1. Basal Excitation, Paired-Pulse Inhibition (PPI), and Short-Term Neuronal Dynamics (STND) in the Dorsal and Ventral Hippocampus

**Figure 1:**
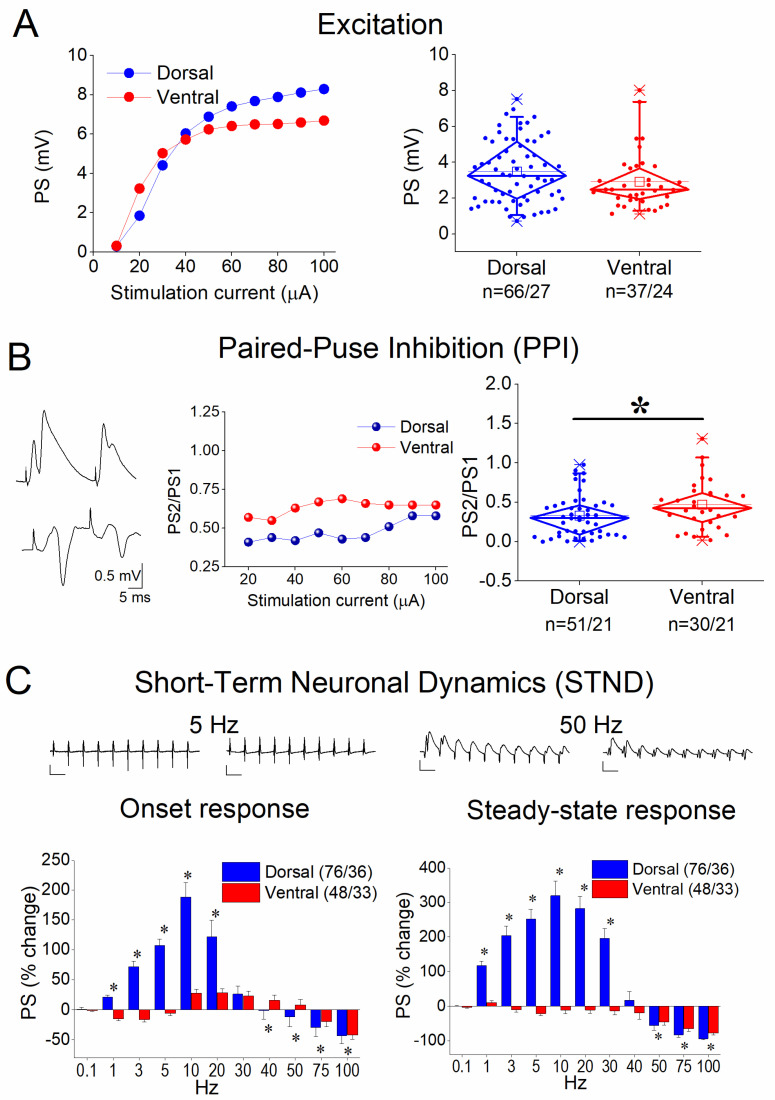
Baseline measurements of excitation (**A**), paired-pulse inhibition (PPI) (**B**), and short-term neuronal dynamics (STND) (**C**) in the dorsal and ventral hippocampus. (**A**) Examples of input-output curves constructed by plotting PS amplitude as a function of stimulating current intensity (left graph) and boxplots of the average PSs calculated from input–output curves for the dorsal and ventral hippocampus (right graph). (**B**) Example traces illustrating the paired-pulse inhibition (PPI) (left graph), the PS2/PS1 ratio plotted against the stimulation current (middle graph), and the average PS2/PS1 ratio (right graph) are shown for the dorsal and ventral hippocampus. (**C**) STND evoked by a ten-pulse train of varying frequency. Example traces of ten consecutive PSs evoked by frequency stimulation applied at 5 and 50 Hz are shown on the top of the graphs. Calibration bars: 1 mV (all traces); 200 ms in traces of 5 Hz and 20 ms in traces of 50 Hz. Asterisks denote differences between the dorsal and the ventral hippocampus (independent *t*-test, at *p* < 0.05).

First, we characterized excitation, PPI, and STND in both the dorsal and ventral hippocampus. Our analysis revealed no significant differences between the dorsal and ventral hippocampus in terms of excitation (independent *t*-test, z = −1.574, *p* = 0.115, Figure 1A) and a significantly higher inhibition in the dorsal than in the ventral hippocampus (independent *t*-test, z = −2.062, *p* = 0.039, Figure 1B), as previously reported [55,56,57]. Furthermore, the comparison of frequency stimulation effects suggested distinct response patterns between the dorsal and ventral hippocampus. Specifically, both onset and steady-state responses were strongly facilitated in the dorsal hippocampus at stimulation frequencies of 1–30 Hz but were depressed at 50–100 Hz. In contrast, the ventral hippocampus exhibited only slight facilitation in the onset response at 10–40 Hz and showed depression across almost the entire frequency range (3–100 Hz). Consequently, steady-state responses were significantly facilitated at 1–30 Hz only in the dorsal hippocampus. Notably, the depression observed at higher frequencies (50–100 Hz) was substantially greater in the dorsal than in the ventral hippocampus. For statistical comparisons between the two hippocampal segments, see Figure 1. These results are in agreement with previous reports [47,54] and highlight a region-specific divergence in short-term neuronal dynamics.

### 3.2. ACEA Enhances Network Excitation and Reduces PPI in the Dorsal but Not the Ventral Hippocampus

**Figure 2:**
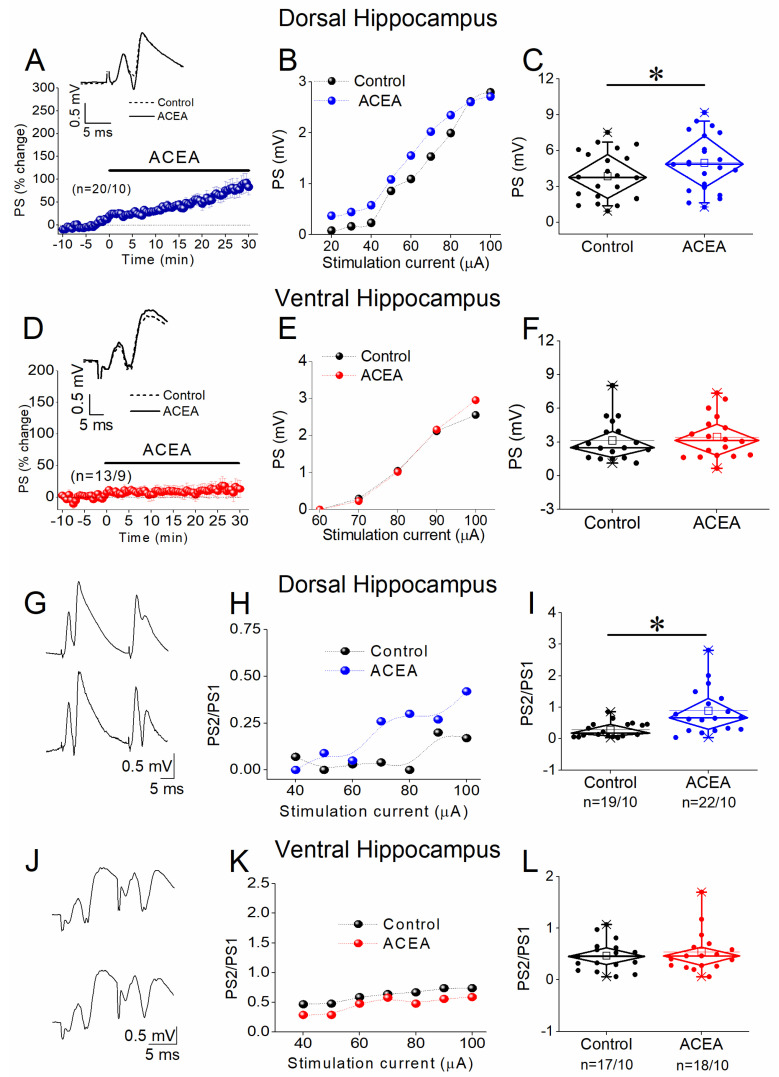
Effects of ACEA on excitation and PPI in the dorsal and ventral hippocampus. (**A**) Time course of PSs recorded from the dorsal hippocampus illustrating the enhancing effect of 5 nM ACEA. Example traces are shown in the insert. (**B**) Example of input–output curves between PS and stimulation current intensity (PS/I) in the dorsal hippocampus before and after ACEA application. Note the drug-induced upper-left shift of the curve. (**C**) Box plot of the average PSs under control and drug conditions. (**D**) Time course of PSs recorded from the ventral hippocampus before and during ACEA application; example traces are shown on the top of the graph. (**E**) Example of PS/I curves in the ventral hippocampus before and after ACEA application. (**F**) Box plot of the average PSs under control and drug conditions. (**G**) Example traces of PSs evoked by paired-pulse stimulation in a dorsal hippocampal slice illustrating that ACEA enhances the ratio between PS2 and PS1 (PS/PS1). (**H**) Graph of PS2/PS1 ratio plotted against stimulation current intensity before and during ACEA application in the dorsal hippocampus. Note that ACEA produces an upward shift of the ratio, indicating a reduction in PPI. (**I**) Box plot of the average PS2/PS1 ratio in the dorsal hippocampus. (**J**) Example traces of PSs evoked by paired-pulse stimulation in a ventral hippocampal slice showing that ACEA did not significantly affect the PS/PS1 ratio. (**K**) Graph of the PS2/PS1 ratio as a function of stimulation current intensity before and during ACEA application in the ventral hippocampus. (**L**) Box plot of the average PS2/PS1 ratio in the ventral hippocampus. Asterisks denote a statistically significant drug effect at *p* < 0.05 (paired *t*-test).

We examined the effects of the CB1 receptor agonist ACEA on network excitation (PS), PPI, and STND in both hippocampal segments (Figure 2). We found that 5 nM ACEA significantly increased the PSs in the dorsal hippocampus (paired *t*-test, t = −3.355, *p* = 0.003, Figure 2A–C) but not the ventral hippocampus (paired *t*-test, z = −1.154, *p* = 0.248, Figure 2D–F). Next, we investigated whether the enhancing effect of ACEA on network excitation in the dorsal hippocampus could be due to a depressive drug effect on PPI. We found that the application of ACEA significantly reduced PPI in the dorsal hippocampus (paired *t*-test, z = −3.702, *p* < 0.001, Figure 2G–I) but not the ventral hippocampus (paired *t*-test, z = −0.544, *p* = 0.586, Figure 2J–L). These results suggest that CB1 receptor activation enhances network excitation in the dorsal hippocampus by reducing synaptic inhibition effectiveness, without significantly affecting excitation or inhibition in the ventral hippocampus.

### 3.3. ACEA Modulates STND More in the Dorsal Than in the Ventral Hippocampus

**Figure 3:**
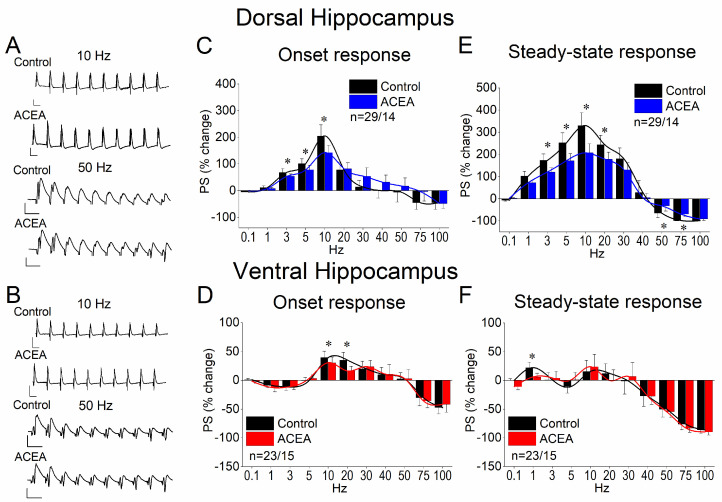
Effects of ACEA on STND in the dorsal (**A**–**C**) and ventral (**D**–**F**) hippocampus. (**A**,**B**) Representative example traces of PSs evoked by a ten-pulse train at 10 and 50 Hz in the dorsal (**A**) and the ventral (**B**) hippocampus. Calibration bars: 1 mV (all traces); 50 ms in traces of 10 Hz and 20 ms in traces of 50 Hz. (**C**,**D**) Effect of 5 nM ACEA on the first conditioned PS (onset response) plotted as a function of the stimulation frequency in the dorsal (**C**) and the ventral (**D**) hippocampus. (**E**,**F**) Effect of 5 nM ACEA on the steady-state response (average of the 8th–10th conditioned responses) plotted as a function of the stimulation frequency in the dorsal (**E**) and the ventral (**F**) hippocampus. Asterisks denote statistically a significant effect of the drug at *p* < 0.05. The number of slices/rats is stated on the graphs.

Subsequently, we investigated how the activation of CB1 receptors by ACEA modulates information flow across the hippocampal CA1 region of the dorsal and ventral hippocampus during repeated activation. We found that ACEA produced significant changes in STND in both segments of the hippocampus. However, the effects of ACEA were pronounced in the dorsal hippocampus but minimal in the ventral hippocampus (Figure 3). More specifically, in the dorsal hippocampus, ACEA reduced the facilitation of the onset response at stimulation frequencies of 3–10 Hz and the facilitation of steady-state response at stimulation frequencies 1–20 Hz (Figure 3A–C). Furthermore, ACEA reduced the frequency depression of the dorsal hippocampus steady-state response at 50–75 Hz (Figure 3C). The drug’s effects on the ventral hippocampus were limited to reducing the facilitation of the onset response at 10–20 Hz (Figure 3E). The statistical analysis results of ACEA’s effects on STND are presented in Figure 3. These data suggest that the activation of CB1 receptors by ACEA modulates the flow of information through the CA1 region mainly in the dorsal hippocampus.

### 3.4. WIN55,212-2 Enhances Network Excitation and Reduces PPI in the Dorsal but Not the Ventral Hippocampus

**Figure 4:**
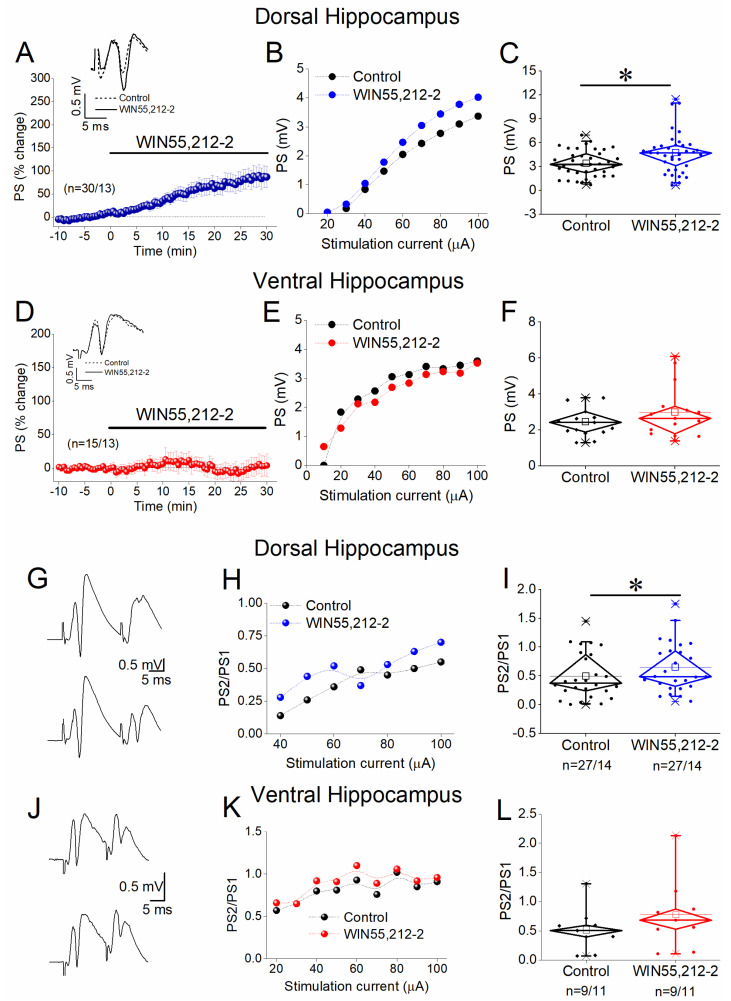
Effects of WIN55,212 on excitation and PPI in the dorsal and ventral hippocampus. (**A**) Time course of PSs in the dorsal hippocampus showing the enhancing effect of 5 μM WIN55,212. (**B**) Example of PS/I curve in the dorsal hippocampus before and after WIN55,212 application. (**C**) Box plot of the average PSs showing the significant drug-induced increase in PSs in the dorsal hippocampus. (**D**) Time course of PSs in the ventral hippocampus. (**E**) Example of PS/I curves in the ventral hippocampus before and after ACEA application. (**F**) Box plot of the average PSs showing the absence of a drug effect on network excitation in the ventral hippocampus. (**G**) Example traces of PSs evoked by paired-pulse stimulation in a dorsal hippocampal slice illustrating that the application of WIN55,212 enhances the PS/PS1 ratio. (**H**) Graph of the PS2/PS1 ratio plotted against stimulation current intensity in the dorsal hippocampus. (**I**) Box plot of the average PS2/PS1 ratio in the dorsal hippocampus showing the significant drug-induced decrease in PPI indicated by the increase in the PS2/PS1 ratio. (**J**) Example traces of PSs evoked by paired-pulse stimulation in a ventral hippocampal slice. (**K**) Graph of the PS2/PS1 ratio as a function of stimulation current intensity in the ventral hippocampus. (**L**) Box plot of the average PS2/PS1 ratio in the ventral hippocampus showing that WIN55,212 does not significantly affect PPI in the ventral hippocampus. Asterisks denote statistically significant drug effect at *p* < 0.05 (paired *t*-test).

Given the significant effects of ACEA on the physiology of the dorsal hippocampus alone, we sought to examine the impact of CB receptor activation using a second agonist, WIN55,212-2. We applied 5 μM WIN55,212-2 to dorsal and ventral hippocampal slices and quantified its effects on PS, PPI, and STND. The results we found were like those for ACEA. Specifically, we found that WIN55,212-2 significantly increased the PSs in the dorsal hippocampus (paired *t*-test, z =−3.870, *p* < 0.001; Figure 4A–C) but not in the ventral hippocampus (paired *t*-test, t = −1.420, *p* = 0.193; Figure 4D–F). The drug effect on the network excitation of the dorsal hippocampus was associated with a significant reduction in PPI (paired *t*-test, z = −2.349, *p* = 0.019, Figure 4G–I). In contrast, WIN55,212-2 did not significantly affect PPI in the ventral hippocampus (paired *t*-test, t = −1.42, *p* = 0.193; Figure 4J–L). These results suggested that the suppression of synaptic inhibition induced by WIN55,212-2 may underlie the enhancement of network excitation in the dorsal hippocampus.

### 3.5. WIN55,212-2 Regulates STND More in the Dorsal Than in the Ventral Hippocampus

**Figure 5:**
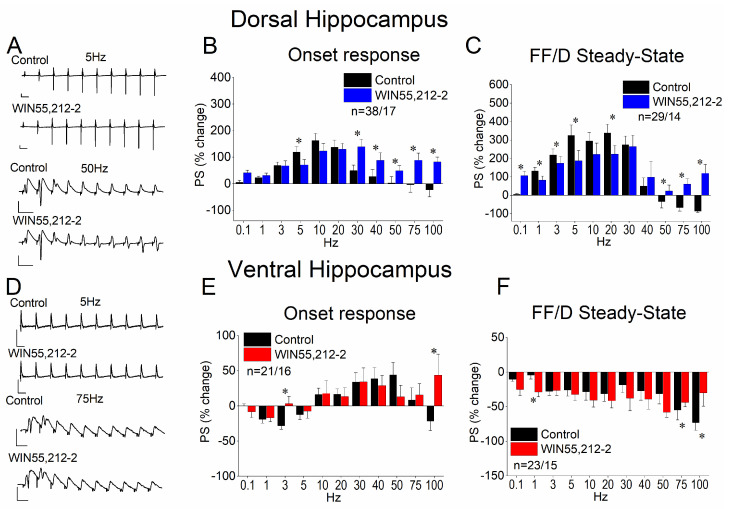
Effects of WIN55,212 on STND in the dorsal (**A**–**C**) and ventral (**D**–**F**) hippocampus. (**A**,**D**) Representative example traces of PSs evoked by a ten-pulse train at 5 and 50 Hz in the dorsal (**A**) 5 and 75 Hz and the ventral (**D**) hippocampus. Calibration bars: 1 mV (all traces); 100 ms in traces of 5 Hz, 20 ms in traces of 50, and 10 ms in traces of 75 Hz. (**B**,**E**) Effect of 5μM WIN55,212 on the first conditioned PSs (onset response) plotted as a function of stimulation frequency in the dorsal (**B**) and the ventral (**E**) hippocampus. (**C**,**F**) Effect of 5 μM WIN55,212 on the steady-state response (average of the 8th–10th conditioned responses) plotted as a function of the stimulation frequency in the dorsal (**C**) and the ventral (**F**) hippocampus. Asterisks denote statistically significant effects of the drug at *p* < 0.05. The number of slices/animals is stated on the graphs.

Then, we examined possible effects of WIN55,212-2 on STND in the two hippocampal segments. We found that WIN55,212-2 significantly modulated both the onset and the steady-state response in the dorsal hippocampus (Figure 5A–C). More notably, the drug’s effects were most pronounced within the 30–100 Hz stimulation frequency range. Specifically, WIN55,212-2 strongly enhanced the frequency facilitation of the onset response at 30–50 Hz and reversed its depression at 75–100 Hz, leading to robust facilitation (Figure 5B). A similar shift from depression to facilitation was observed in the steady-state response at 50–100 Hz, while frequency facilitation at lower frequencies (1–20 Hz) was diminished. The effects of WIN55,212-2 on the ventral hippocampus were considerably less pronounced (Figure 5D–F). Specifically, the drug reversed the depression of the onset response into facilitation at stimulation frequencies of 3 and 100 Hz (Figure 5E). Additionally, WIN55,212-2 attenuated the depression of the steady-state response at 75–100 Hz (Figure 5F). The results of the statistical analysis are presented in Figure 5.

### 3.6. CB1 Receptors Are Similarly Expressed in the Dorsal and Ventral CA1 Hippocampal Regions

**Figure 6:**
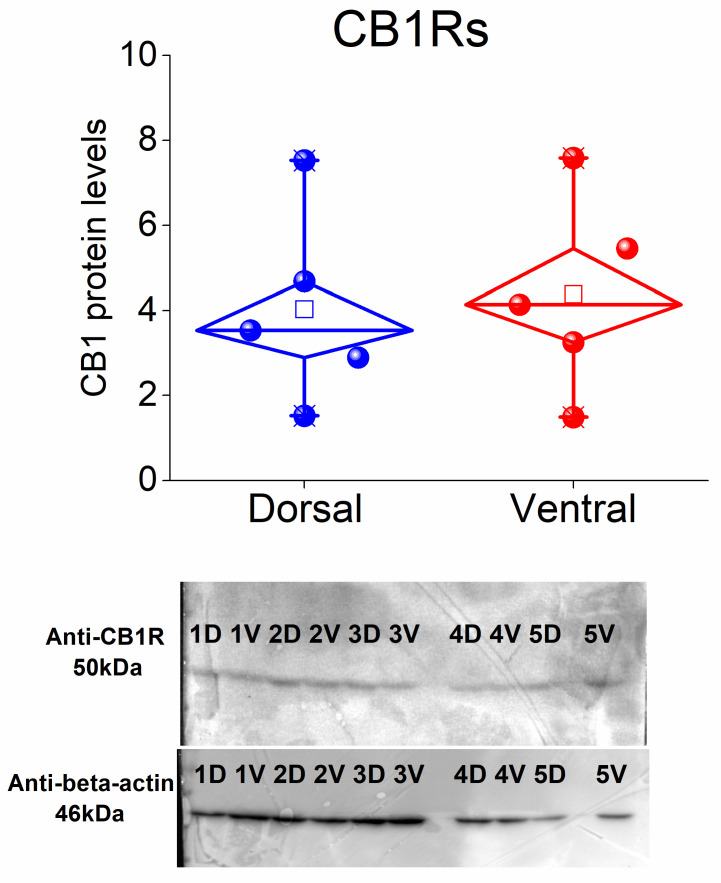
Protein expression of CB1 receptors in dorsal and ventral CA1 hippocampal fields. The protein levels of CB1 receptors in the CA1 field are similar in dorsal and ventral hippocampal regions. Data were collected from five rats.

Given the distinct dorsoventral effects of the two CB1 receptor agonists, we hypothesized that these differences might be attributed to variations in CB1 receptor expression between the two hippocampal segments. To investigate this, we performed a Western blot analysis to compare CB1 receptor levels between the two hippocampal segments. Our findings revealed no statistically significant difference in CB1 receptor expression between the dorsal and ventral CA1 regions (paired *t*-test, t = −0.31, *p* = 0.772; Figure 6 and Supplementary Materials). These results suggest that the differential effects of CB1 receptor agonists in the dorsal and ventral hippocampus are unlikely to be attributed to quantitative differences in receptor expression between the two hippocampal segments. Instead, these differences may stem from variations in the cellular distribution of the receptors or distinct downstream signaling pathways.

### 3.7. Blockade of GIRK Channels Occludes the Effects of WIN55,212-2 on STND

**Figure 7:**
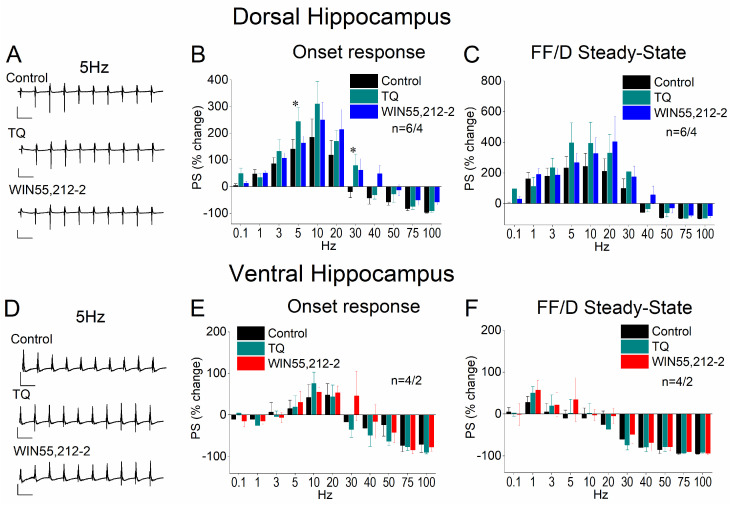
Effects of Tertiapin-Q (TQ, 50 nM) application and subsequent WIN55,212 (10 μM) application on STND in the dorsal (**A**–**C**) and ventral (**D**–**F**) hippocampus. (**A**,**B**) Representative example traces of PSs obtained under the three pharmacological conditions at 5 Hz. Calibration bars: 1 mV; 200 ms. The drug effects are shown for the onset response (**C**,**D**) and the steady-state response (**E**,**F**), in the two segments of the hippocampus. Asterisks denote statistically significant effect of the drug at *p* < 0.05. The number of slices/animals is stated into graphs.

In addition to their well-established presynaptic actions, CB1 receptors can modulate neuronal excitability and, consequently, STND through postsynaptic mechanisms. A recognized postsynaptic target of CB receptors is GIRK channels, the activity of which CB1 receptors inhibit. To investigate this further, we applied WIN 55,212-2 in a set of dorsal hippocampal slices in the presence of the GIRK channel blocker Tertiapin-Q. We found that Tertiapin-Q effectively blocked the effects of WIN55,212-2 on STND in both the dorsal (Figure 7A–C) and ventral hippocampus (Figure 7D–F), suggesting that the CB1 receptor-mediated modulation of STND involves the suppression of GIRK channel activity.

## 4. Discussion

In this study we compared the effects of two agonists of CBs on the local network excitation and the short-term neuronal dynamics, i.e., the dynamic response of the local circuit of the CA1 region, in the dorsal and ventral segment of the hippocampus. We also examined the possible involvement of GIRK channels in the modulation of STND by CBs, and we quantified the level of CBs in both hippocampal segments. The main findings of the present study are the following: (1) The activation of CB1 receptors by ACEA or WIN55,212-2 significantly enhances excitation and reduces paired-pulse inhibition in the CA1 circuitry in the dorsal hippocampus but does not significantly change excitation or inhibition in the ventral hippocampus. (2) The activation of CB1 receptors by ACEA or WIN55,212-2 modulates STND more in the dorsal than in the ventral CA1 hippocampal region. (3) CBRs are similarly expressed in the dorsal and ventral hippocampal CA1 regions. (4) GIRK channels mediate most of the effects of CB1 receptors on STND.

This is the first study providing comparative results about the actions of CB receptors on baseline hippocampus physiology under normal conditions. Furthermore, it is the first study to examine the effects of CB receptors on STND. CB1 receptors acting through Gi/o- and Gq/11-dependent signaling pathways regulate synaptic transmission and neuronal excitability [6,7,8,11]. The main cannabinoid actions in the hippocampus are a suppression of inhibitory and, secondarily, excitatory synaptic transmission through presynaptic CB1 receptors [21,22,23,24,25]; but see also [58]. By these actions, CB1 receptors modulate the excitation/inhibition balance of the local neuronal circuitry [25,26,27].

In the CA1 region, CB1 receptors are predominantly expressed on the terminals of cholecystokinin-containing GABAergic interneurons [59], which belong to basket cells that exert strong inhibitory control over pyramidal cell excitation [60]. The activation of presynaptic CB1 receptors on GABAergic terminals leads to disinhibition and postsynaptic neuron excitation [8]. Accordingly, the concurrent enhancement of CA1 network excitation and the reduction in paired-pulse inhibition observed following CB1 receptor agonist administration may be attributed to decreased excitation of GABAergic interneurons. A similar mechanism could underly the effects of CB1 receptor activation on STND. Specifically, it has been previously shown that inhibition limits the frequency facilitation and enhances the frequency depression of PSs in the hippocampus, thereby shaping the STND profile induced by repetitive stimulation [47]. Thus, it is reasonable to propose that the enhancing effects of CB1 receptor activation on STND are mediated by reduced interneuron excitability.

Interestingly, we found that the blockade of GIRK channels largely occludes the effects of the activation of CB1 receptors on STND in the dorsal hippocampus. This finding is in keeping with previously reported evidence showing that WIN55,212-2 activates GIRK channels [61,62,63] and suggests that at least partly, the effects of WIN55,212-2 on STND are mediated through GIRK channels. Indeed, emerging research indicates that postsynaptic CB1 receptors can influence neuronal excitability through the activation of GIRK channels, leading to the hyperpolarization of neurons [64]. This action is linked to a more general principle that Gi/o-coupled receptors, like opioid, GABA_B_, and muscarinic receptors, activate GIRK channels to hyperpolarize neurons [65,66]. Yet the effect of WIN55,212-2 found here is excitatory rather than inhibitory. Accordingly, the involvement of GIRK channels in the action of WIN55,212-2 on STND could more plausibly be explained by assuming a co-localization of CB1 receptors and GIRK channels on inhibitory interneurons. Indeed, it has been shown that a train of action potentials, at a frequency similar to that used in the present study, induces the endocannabinoid-mediated activation of GIRK channels, resulting in the hyperpolarization and reduced excitability of inhibitory cortical interneurons [43]. Therefore, the occlusion of WIN55,212-2 effects on STND by Tertiapin-Q may be attributed to the cancellation of CB1 receptor-mediated suppression of interneuron activity. Furthermore, the observation that CB1 receptors affect CA1 neuronal circuitry more prominently in the dorsal than in the ventral hippocampus is consistent with the higher expression of GIRK channels in the dorsal CA1 region compared to the ventral counterpart [67,68]. These findings underscore the complex modulation of neuronal circuits by the endocannabinoid system and reveal its distinct role in regulating local neuronal networks along the longitudinal axis of the hippocampus under physiologically relevant conditions.

A main conclusion of the present study is that, under physiologically relevant basal experimental conditions, the dorsal hippocampus appears to be more sensitive to CB1 receptor manipulation, despite the similar expression levels of CB1 receptors in both hippocampal segments. As discussed earlier, this may be partly attributed to the higher expression of GIRK channels in the dorsal compared to the ventral hippocampus, given that these channels appear to mediate a significant portion of CB1 receptor-mediated cellular effects and the expression of CB1 is similar between the two hippocampal segments. However, a more thorough investigation of CB1 receptor expression in terms of cell-type specificity, subcellular localization, and receptor functionality is required to draw definitive conclusions about their involvement in the dorsoventral differences observed in the actions of their agonists. Additionally, based on the present results, it is not possible to determine whether presynaptic or postsynaptic CB1 receptors are involved in the differential effects of their agonists between the two segments of the hippocampus.

### Implications for Functional Specialization Along the Hippocampus

The differential effects of CB1 receptor activation in the dorsal and ventral hippocampus observed in this study extend previous findings on their distinct roles in behavior, memory, and emotional regulation. The dorsal hippocampus, critical for spatial memory and cognition [69,70], is highly sensitive to CB1 receptor-mediated modulation, consistent with reports that cannabinoid administration into the dorsal, but not ventral, hippocampus disrupts tasks such as radial arm maze completion [71] and delays match-to-sample paradigms [72], which crucially involve the dorsal hippocampus [73]. Mechanistically, CB1 receptor-mediated disinhibition and increased excitation leading to altered E/I balance in the dorsal hippocampus likely contribute to the memory deficits induced by cannabinoid exposure [29,71] by impairing precise memory encoding [74]. Furthermore, given the proposed role of short-term synaptic plasticity in memory formation [75,76], the dorsal hippocampus’s sensitivity to the CB1 receptor-mediated modulation of STND could help explain the vulnerability of spatial memory to acute cannabinoid exposure.

In contrast, the ventral hippocampus, key for emotional processing and stress regulation [77,78,79,80], showed minimal baseline sensitivity to CB1 receptor activation. Interestingly, a dorsoventral differential role of CB1 receptors has been documented in stress and anxiety regulation. Acute stress, for example, leads to the upregulation of CB1 receptors in the dorsal hippocampus of stress-vulnerable rats, potentially exacerbating network disinhibition and instability, whereas it leads to a downregulation of CB1 receptor expression in the ventral hippocampus [81]. Additionally, low doses, but not high doses, of cannabinoids administered into the ventral hippocampus induce an anxiolytic-like response in elevated plus maze tests [82], whereas the administration of WIN55,212-2 into the dorsal hippocampus induces anxiogenic-like effects [83].

These findings imply that chronic stress could predispose the dorsal hippocampus to cognitive deficits through CB1 receptor dysregulation, while the resistance of the ventral hippocampus may represent an adaptive mechanism to preserve network stability under stress, consistent with its inherent tendency to hyperexcitability [34] and its role in hypothalamic–pituitary–adrenal (HPA) axis modulation [26]. Thus, while cannabinoid signaling at rest may be limited in the ventral hippocampus, it may become more dynamically engaged during stress to modulate anxiety-related behaviors.

Previous observations have suggested a potential protective role of CB1 receptors in the dorsal hippocampus during intense neuronal activation. Specifically, CB1 receptors appear to limit the suppression of excitatory transmission and, more notably, the suppression of inhibitory synaptic transmission. This action helps to prevent the marked increase in network excitability observed in the CA1 region following the co-activation of NMDA and metabotropic glutamate receptors [35].

The present findings, along with previous work, suggest that cannabinoid actions vary significantly along the hippocampal axis depending on the physiological or behavioral context. Cannabinoids impair dorsal hippocampus-mediated cognition even under baseline conditions, whereas their impact on ventral hippocampus-related emotional behaviors emerges primarily under stress. Thus, while dorsal CB1 receptor activation contributes to cognitive vulnerability, ventral CB1 receptor signaling may be tuned to buffer stress. This region-specific functional dichotomy may lead to a challenge regarding therapeutic implications, highlighting the need for region-specific approaches. Thus, future strategies should aim to selectively mitigate CB1 receptor activation in the dorsal hippocampus to prevent memory impairments without compromising the potentially beneficial anxiolytic effects mediated by ventral hippocampal CB1 receptors. Overall, understanding the regional specialization of endocannabinoid signaling is crucial for the development of targeted interventions in cannabinoid-related cognitive and emotional disorders.

## 5. Conclusions

This study demonstrates that the endocannabinoid system, via CB1 receptor activation, modulates hippocampal network dynamics in a region-specific manner. Specifically, we show that CB1 receptor agonists (ACEA and WIN55,212-2) significantly enhance excitability, reduce paired-pulse inhibition, and alter short-term neuronal dynamics predominantly in the dorsal hippocampus, with minimal effects on the ventral region. These effects are not apparently attributable to differences in CB1 receptor expression, which is comparable between hippocampal segments, but likely arise from downstream signaling mechanisms, particularly those involving GIRK channels. These findings offer novel insights into the heterogeneity of endocannabinoid signaling and its implications for the modulation of information processing in the hippocampus and also contribute to a deeper understanding of the functional differentiation along the dorsoventral axis of the hippocampus. This functional differentiation may help explain the distinct effects of cannabinoids on memory- and emotion-related behaviors, which are primarily mediated by the dorsal and ventral hippocampus, respectively. Future studies could more directly investigate the mechanisms underlying this signaling diversity and its behavioral correlates. Understanding the modulation of hippocampal circuitry and functions by the endocannabinoid system may be essential for the development of targeted cannabinoid-based interventions for neurological and psychiatric disorders.

## Data Availability

All the datasets generated and analyzed during this study are kept in the Physiology Lab, Dept of Medicine, University of Patras, and they are available from the corresponding author on reasonable request.

## Supplementary Materials

The following supporting information can be downloaded at: https://www.mdpi.com/article/10.3390/biology14060642/s1, Supplementary Figure S1: Original Western blot image showing the protein lander (on the left) and bands for CB1 receptor protein and β-actin. The samples corresponding to those presented in Figure 6 are indicated within the frame; Supplementary Figure S2: Graphs showing the results of frequency stimulation under control conditions (Control), and under drug condition before (non-adjusted) the after adjusting the PS to control levels (adjusted). Data were obtained from the dorsal hippocampus where CB1 receptor agonists (ACEA, A-B and WIN55,212-2, C-D) produced an increase in PS.

## Abbreviations

The following abbreviations are used in this manuscript:

CB	Cannabinoid
PS	Population spike
PPI	Paired-pulse inhibition
STND	Short-term neuronal dynamics
E/I	Excitation/inhibition
GIRK	Inwardly rectifying potassium
