# Supplementary material for: Cannabinoid Modulation of Excitability and Short-Term Neuronal Dynamics in the Dorsal and Ventral Hippocampus
Source: Biology (Basel). 2025 May 31;14(6):642. doi: 10.3390/biology14060642 (PMC12189292; doi:10.3390/biology14060642)
Supplement: Supplementary file 1 [file biology-14-00642-s001.zip › biology-3601470-supplementary.pdf]

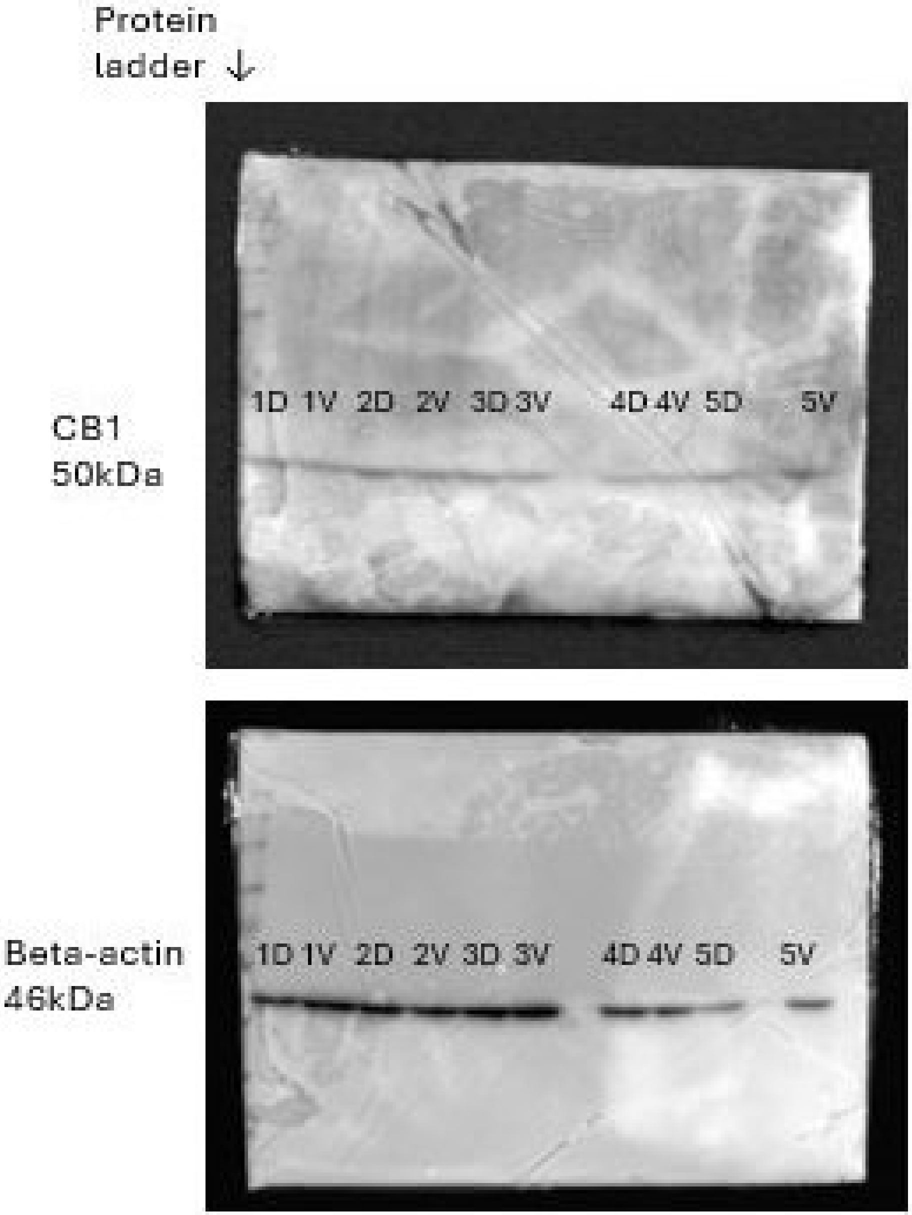

Supplementary Figure S1. Original Western blot image showing the protein lander (on the left) and bands for CB1 receptor protein and β-actin. The samples corresponding to those presented in Figure 6 are indicated within the frame.

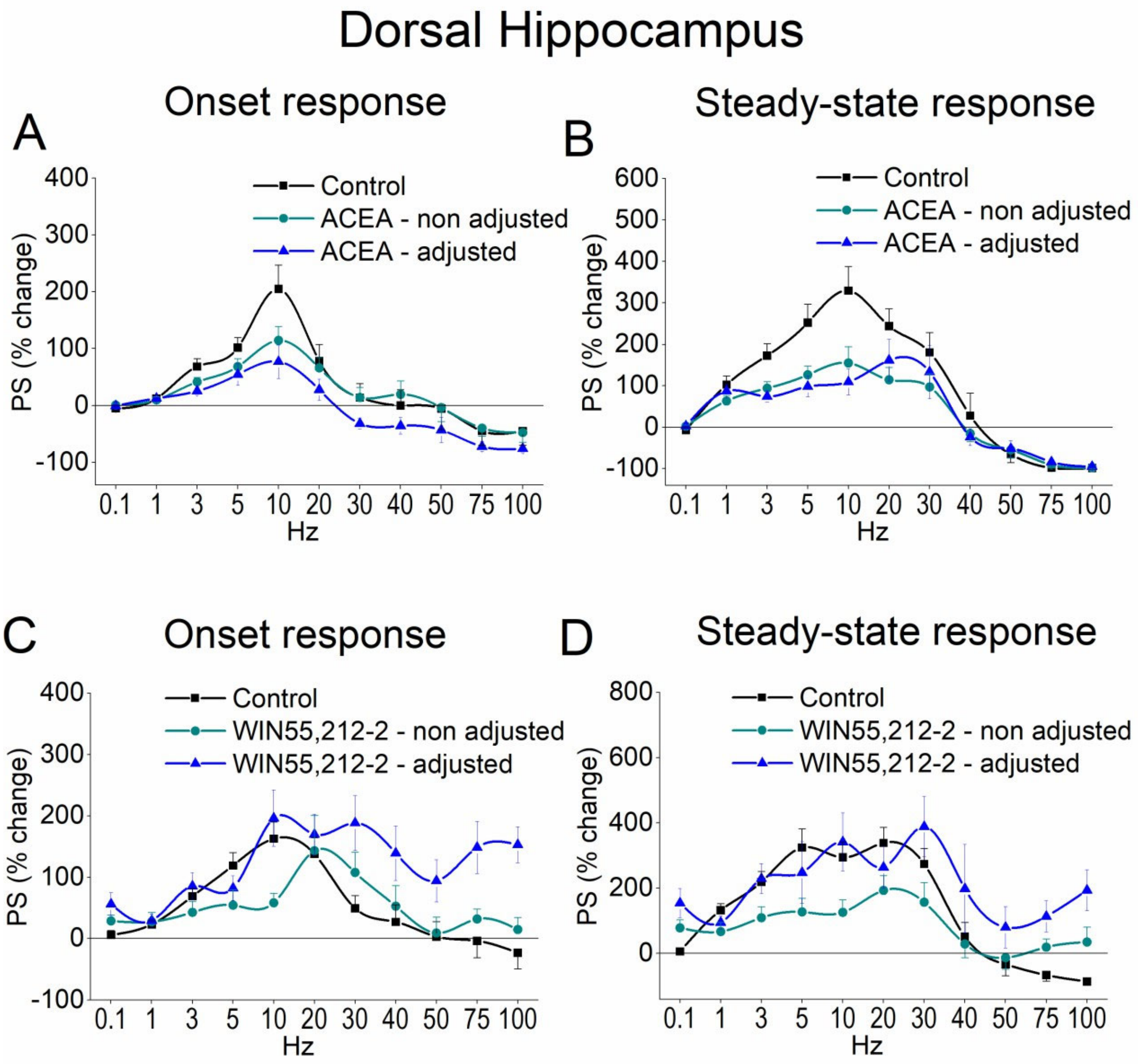

Supplementary Figure S2. Graphs showing the results of frequency stimulation under control conditions (Control), and under drug condition before (non-adjusted) the after adjusting the PS to control levels (adjusted). Data were obtained from the dorsal hippocampus where CB1 receptor agonists (ACEA, A-B and WIN55,212-2, C-D) produced an increase in PS.
